# Data profile: cancer sample cohorts (stomach, breast, colorectal, and liver) in Korea

**DOI:** 10.4178/epih.e2025058

**Published:** 2025-10-14

**Authors:** Daewoo Pak, Suk Yong Jang, Jin-Ha Yoon, Dong Wook Kim, Jin-Won Noh, Dong-Woo Choi, Minyeong Guk, Hyeri Kim, Ju-Won Oh, Heejung Chae, Hyun-Joo Kong, Gi Hyun Kim, Ji Woong Nam, Ga Ram Lee, Dayun Park, Jehoo Jeon, Byungyoon Yun, Ki-Bong Yoo, Kui Son Choi

**Affiliations:** 1 Division of Data Science, Yonsei University Mirae Campus, Wonju, Korea; 2 Institute of Health Services Research, Yonsei University, Seoul, Korea; 3 Department of Healthcare Management, Graduate School of Public Health, Yonsei University, Seoul, Korea; 4 The Institute for Occupational Health, Yonsei University College of Medicine, Seoul, Korea; 5 Department of Preventive Medicine, Yonsei University College of Medicine, Seoul, Korea; 6 Institute for Innovation in Digital Healthcare, Yonsei University Health System, Seoul, Korea; 7 Department of Information and Statistics, Gyeongsang National University, Jinju, Korea; 8 Department of Bio & Medical Big Data, Research Institute of Natural Science, Gyeongsang National University, Jinju, Korea; 9 Division of Health Administration, Yonsei University Mirae Campus, Wonju, Korea; 10 Institute for Planetary Health, Yonsei University, Wonju, Korea; 11 Cancer Data Center, National Cancer Control Institute, National Cancer Center, Goyang, Korea; 12 Center for Breast Cancer, Hospital, National Cancer Center, Goyang, Korea; 13 Health Insurance Research Institute, National Health Insurance Service, Wonju, Korea; 14 Department of Health Administration, Yonsei University Mirae Campus, Wonju, Korea; 15 Department of Eligibility and Imposition Eastern Part Cheongju, National Health Insurance Service, Cheongju, Korea; 16 Department of Image Processing Algorithm Development, Imaging R&D Center, Osstem implant Co., Ltd., Seoul, Korea; 17 AI Part, Planning AI Division, New Power Plasma Co., Ltd., Suwon, Korea; 18 Graduate School of Cancer Science and Policy, National Cancer Center, Goyang, Korea

**Keywords:** Data profile, Breast neoplasms, Stomach neoplasms, Liver neoplasms, Colorectal neoplasms

## Abstract

Cancer Public Library Database (CPLD) links data from four major population-based public sources: the Korea National Cancer Incidence Database in the Korea Central Cancer Registry, cause-of-death data in Statistics Korea, the National Health Information Database in the National Health Insurance Service, and the National Health Insurance Research Database in the Health Insurance Review & Assessment Service. The National Cancer Data Center has developed a new nationally representative sample cohort dataset from Korean Clinical Data Utilization for Research Excellence project (K-CURE) CPLD: Stomach Cancer Sample Cohort, Breast Cancer Sample Cohort, Colorectal Cancer Sample Cohort, and Liver Cancer Sample Cohort. The sample populations consisted of approximately 21% of all cancer patients from 2012 to 2019. The populations of the Stomach Cancer Sample Cohort, Breast Cancer Sample Cohort, Colorectal Cancer Sample Cohort, and Liver Cancer Sample Cohort were 51,951, 39,586, 53,485, and 27,375 patients, respectively. The dataset included cancer incidence information, demographics, socioeconomic variables, health utilization data (procedures, diagnoses, and medications), general health checkup data, cancer screening data before and after the cancer incidence, as well as death information. These cohorts could help researchers analyze time-to-event data on mortality, treatment outcomes, comorbid conditions following a cancer diagnosis, and cancer incidence risk factors. The data can be accessed through the K-CURE portal (https://k-cure.mohw.go.kr/).

## INTRODUCTION

The need for samples and customized databases is emerging in Korea with the increasing demand for public health big data [1-3]. The frequently used national health insurance big data includes the entire population, making it an excellent data source for representativeness and sample size [4]. In Korea, this data source comprises three institutions: the Health Insurance Review & Assessment Service (HIRA), the National Health Insurance Service (NHIS), and Statistics Korea. HIRA encompasses health utilization, including expenditures, length of stay, encounter types, procedure codes, diagnosis information, and medications. NHIS manages insurance eligibility and health examination data, while Statistics Korea comprises cause-of-death data.

The HIRA and NHIS have released various sample cohort data [4-8]. However, these datasets include health utilization information based on only claims data. They lack detailed clinical information and laboratory tests and have limitations regarding coding accuracy [9]. Additionally, these data sources cannot identify the cancer stage and detailed topography. In contrast, the Korea Central Cancer Registry (KCCR) managed by the National Cancer Center (NCC) consists of staging and detailed clinical information on cancer, offering reliable data accuracy [10]. However, it lacks data on the socioeconomic status and healthcare utilization in patients with cancer [11-13].

Consequently, combining these databases can complement their strengths and weaknesses. The National Cancer Data Center (NCDC) of the NCC developed a novel database called the Cancer Public Library Database (CPLD) under the Korean Clinical Data Utilization for Research Excellence project (K-CURE) [14]. The NCDC began providing CPLD data for research purposes on demand in 2023. The customized data based on demand can satisfy various research topics; however, its use is restricted due to de-identification issues and security concerns. Therefore, researchers are required to visit designated offline research centers to access the data. To address this inconvenience, the NCDC has developed and opened sample cohorts for stomach, breast, colorectal, and liver cancers to promote cancer research with a simple and fast administration process and a remotely accessible environment.

## DATA RESOURCE AND POPULATION COVERAGE OF THE CANCER SAMPLE COHORTS

**Figure 1.**
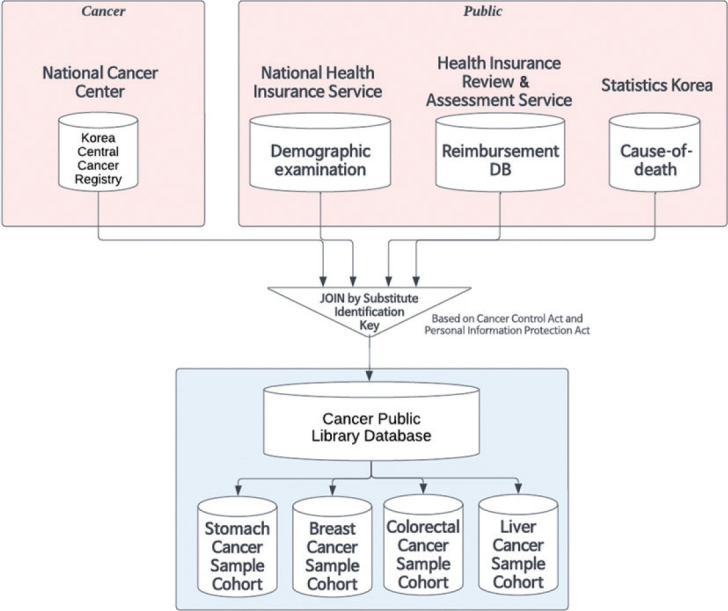
Data structure of Cancer Public Library Database and four sample cohorts.

All cancer sample cohorts were derived from the CPLD, which links data from the KCCR, HIRA health insurance claim data, NHIS insurance eligibility and examination data, and cause-of-death data of Statistics Korea (Figure 1). As a cancer registry data, KCCR has included all patients diagnosed with cancer or treated in hospitals since 1999 [15]. To merge data from multiple institutes, linkage tables for the Substitute Identification Key were generated for internal integration and de-identification across the four institutions. The Substitute Identification Key is a series of values assigned by the data-providing institutions to uniquely identify individuals. Per the Personal Information Protection Act and the Cancer Control Act [16], the data-providing institutions share de-identified personal information after anonymization upon requesting data from the NCDC.

We identified the target population to construct nationally representative cohorts as follows. Among patients with stomach cancer, breast cancer, colorectal cancer, and liver cancer in KCCR data, we excluded secondary cancers and missing data for the age, sex, region, year of diagnosis, cancer type, and cancer stage. To protect privacy, if there were fewer than five individuals within a strata defined by cancer diagnosis year, sex, age at diagnosis, region (metropolitan, city, or rural), cancer type, or cancer stage, such strata were excluded from the population. The target population of patients with breast cancer exclusively comprised females. So the target population of all cancer patients registered between 2012 and 2019 included 248,103 patients with stomach cancer, 192,907 with breast cancer, 261,291 with colorectal cancer, and 124,324 with liver cancer.

Stratified random sampling with proportional allocation was used to construct a representative sample cohort for each cancer type from the target population using. Sampling is conducted to extract newly registered patients with cancer every year. The population units were partitioned into strata defined by the year of diagnosis, sex, age at diagnosis, region, and Surveillance, Epidemiology, and End Results (SEER) summary stage (in situ, localized, regional, distant, and unknown). Additionally, a probability sample of units was selected from each stratum, while ensuring the representativeness of the total annual medical costs within the stratum. Owing to the small strata for patients aged <39 years or >80 years diagnosed with cancer, the age at diagnosis for stratification was recategorized into six groups as follows: <39 years, 10-year intervals between 40 years and 79 years, and ≥80 years. The region was categorized into 17 groups, namely, Seoul, Busan, Daegu, Incheon, Gwangju, Daejeon, Ulsan, Sejong, Gyeonggi, Gangwon, Chungbuk, Chungnam, Jeonbuk, Jeonnam, Gyeongbuk, Gyeongnam, and Jeju, for sampling stratification. However, it was recategorized into three groups: metropolitan, city, and rural, for public sample data because of de-identification. We constructed 3,117 strata for breast cancer, 6,021 for stomach cancer, 5,218 for liver cancer, and 6,296 for colorectal cancer.

In each stratum, samples were drawn at a sampling rate of 20%, much larger than the theoretically derived sizes required to achieve a 1% margin of error for the mean of total annual medical expenses. Due to de-identification, the sampling rates were limited to approximately 20%. Their representativeness was assessed by determining whether the population averages of the stratum fell within a 95% confidence interval (CI) for sampling without replacement, which is defined as


$$CI={\bar{y}}_{h}\pm 1.96\sqrt{\frac{N_{h}-n_{h}}{N_{h}-1}}\frac{\sigma_{h}}{\sqrt{n_{h}}},$$

where, for the *h*-th stratum, ${\bar{y}}_{h}$ is the sample mean, *N_h_* and *n_h_* are the population and sample sizes, respectively, and *σ_h_* is the known population standard deviation. If the representativeness was not achieved for a stratum, independent random sampling was conducted up to 50 times to generate candidate sets of samples for the stratum. Among those ensuring representativeness, the samples with the mean closest to the population mean of the stratum were selected as the final samples. If the initial sample size was insufficient to achieve representativeness, we incrementally increased the sample size for the stratum and repeated the process until the representativeness was attained.

**Figure 2.**
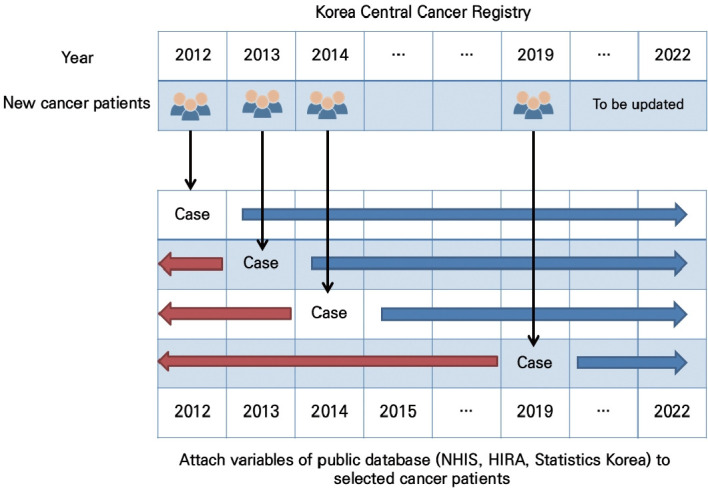
Process of constructing the sample cohort data. NHIS, National Health Insurance Service; HIRA, Health Insurance Review & Assesment Service.

Finally, the samples of the stomach, breast, colorectal, and liver cancer sample cohorts comprised 51,951 (20.9%), 39,586 (20.5%), 53,485 (20.5%), and 27,375 (22.0%) patients, respectively (Table 1). The age-specific distribution of cancer incidence varied across cancer types. Among patients aged 60 years or older, the proportions were 65.2% for stomach cancer, 25.0% for breast cancer, 66.2% for colorectal cancer, and 61.8% for liver cancer, and these age-related incidence patterns were well represented in the sample cohorts. Note that the incidence of stomach, colorectal, and liver cancers increases with age due to the cumulative effects of carcinogenic exposures and biological aging [17], while breast cancer accounts for a lower proportion in older age groups because it more commonly occurs in their 40s and 50s in Asian populations, including Korea [18]. All cancers, except breast cancer, for which the population consisted only of female, showed a higher incidence in male than in female, with male-to-female rate ratios of 2.1 for stomach cancer, 1.5 for colorectal cancer, and 3.0 for liver cancer with corresponding values of 2.0, 1.5, and 2.6 in the sample cohorts, respectively. There were no substantial differences in the distribution of cancer incidence by year of diagnosis across all cancer types during the study period. The variables from the public database were attached to selected patients from 2012 to 2021 (Figure 2). The NCC plans to update the cancer clinical library sample database annually.

The representativeness of the follow-up years cannot be guaranteed; nonetheless, a sufficient sample size for the initial cohort could help ensure representativeness. We assessed and confirmed the representativeness of all strata by examining whether the 95% CI for sampling without replacement consisted of the average total annual medical expenses and statistical power. Table 2 summarizes the frequencies and medical costs by cancer type and age group. Other stratification variables were omitted in Table 2. The medical costs in sample cohorts by cancer type and age group accounted for the medical costs of the population. Furthermore, it shows a clear trend of increasing medical costs with advancing age across most cancer types, particularly for stomach and colorectal cancers. Liver cancer exhibited the highest average medical costs across all age groups, while stomach cancer showed relatively lower medical costs compared to other cancer types. Breast cancer had the highest costs on average in the 50-69 age groups. The similarity in average medical costs between the sample cohorts and the population dataset further demonstrates the representativeness of the sample cohorts. These patterns highlight the age-related economic burden of cancer care and underscore the potential utility of this dataset for studies on healthcare resource allocation.

**Figure 3.**
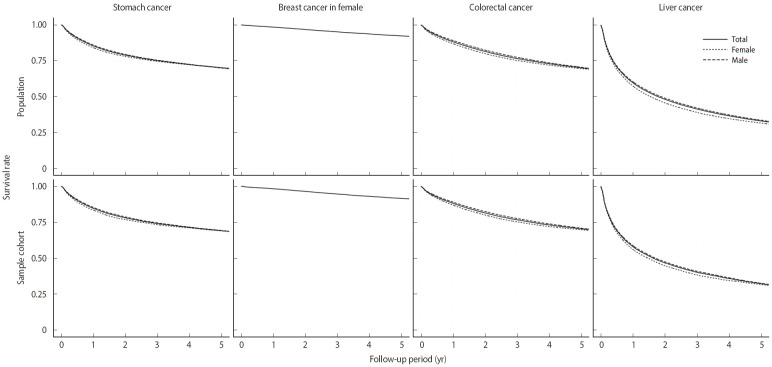
Survival curves of patients by cancer type and sex in the population and sample cohorts..

In Figure 3, Kaplan–Meier survival curves for patients in both the population and sample cohorts are depicted by sex for each cancer type. Both the population and sample cohorts showed similar survival curves. In each stratum, we further evaluated whether the samples adequately represented the survival distribution of the population using a one-sample log-rank test. This test is commonly used to compare the survival curve of a sample with that of a defined reference population [19]. The proportion of strata where the test did not reject the null hypothesis—that the survival of the sample population is the same as that of the corresponding population—at a significance level of 0.05 was 91.1% for stomach cancer, 97.9% for breast cancer in females, 92.7% for colorectal cancer, and 93.8% for liver cancer.

The follow-up times for each cancer type were consistent between the population and the sample cohorts. The median follow-up times for the population (the sample cohort) were 3.5 (3.6) years for breast cancer, 4.0 (4.0) years for colorectal cancer, 4.3 (4.3) years for liver cancer, and 4.1 (4.1) years for stomach cancer. Furthermore, the maximum follow-up times for the population (the sample cohort) were 8.9 (8.8) years for breast cancer, 9.0 (8.9) years for colorectal cancer, 9.0 (8.8) years for liver cancer, and 8.9 (8.7) years for stomach cancer, while the minimum follow-up time was 0 years across all cancer types. We further examined the median follow-up times by age at diagnosis. For cases diagnosed in 2012, the median follow-up time was approximately 7.5 years across all cancer types in both the population and the sample cohorts. In contrast, for cases diagnosed in 2019, the median follow-up times were approximately 0.5 years for breast and stomach cancers, 0.6 years for colorectal cancer, and 0.7 years for liver cancer, with consistent patterns observed in both the population and the sample cohorts.

## MEASURES

Almost the same database was used for all cohorts (Table 3). The information was as follows: KCCR data and cancer screening records (stomach, breast, colorectal, and liver cancers) from the KCCR from 2012 to 2019; health insurance claim data, such as health utilization data, diagnosis records, and prescription details from HIRA from 2012 to 2022; insurance eligibility data and general health checkups from NHIS from 2012 to 2022; and cause-of-death data from Statistics Korea from 2012 to 2021.

Stomach cancer screening included results of an esophagogastroduodenoscopy. Breast cancer screening included results of mammography and tissue biopsy. Liver cancer screening included abdominal ultrasonography and serum alpha-fetoprotein test; colorectal cancer screening included fecal occult blood test for the first test, and colonoscopy (biopsy) or double contrast barium enema for the second test. The K-CURE portal (https://k-cure.mohw.go.kr/) contains all information on the variables [14,20].

In summary, the sample cohort included detailed information on cancer incidence. Additionally, it included demographic and socioeconomic variables, health utilization data (including procedures, diagnoses, and medications), general health checkup data before and after cancer incidence, and death and cancer screening data. More variables in CPLD were present, but those related to administrative purposes were excluded from the sample cohorts.

### Ethics statement

This sampling process was exempt from the Institutional Review Board of Yonsei University Mirae.

## DATA RESOURCE USE

Several studies on machine learning research take a long time to analyze because the sample data is accessible remotely 24 hours a day. In Kang et al. [21]’s paper, a machine learning model was proposed to predict survival of young breast cancer patients. Cancer type was breast cancer defined as International Classification of Diseases, 10th revision (ICD-10) C500-C506, C508, and C509 and primary endpoint was all-cause mortality within 5 years of diagnosis. They concluded random survival forest, gradient boosting survival analysis, extra survival trees, and penalized Cox probability hazard lasso using Tensorflow version 1.15.5 and Python version 3.7.5. The highest area under the curve (AUC) were 0.87 in young patients and 0.83 in elderly patients by the extra survival trees model.

Kang et al. [22] published a study on mortality and gastric cancer. This research used various machine learning including extensions of gradient boosting machine models and random forests. Gastric cancer was defined as C16 in ICD-10 and the outcomes were the all-cause mortality and disease-specific (gastric cancer) mortality. The highest AUCs were 0.795 for all-cause mortality using the gradient boosting machine and 0.867 for disease-specific mortality using the light gradient boosting machine. This study used R version 3.8.1 (R Foundation for Statistical Computing, Vienna, Austria), Python version 3.8.10, Optuna version 3.3.0.

These two recent studies [21,22] demonstrate that researchers can conduct diverse prognostic analyses on cancer incidence cases by leveraging detailed cancer-related data and advanced analytical environments. While the NHIS National Sample Cohort [6] poses challenges for implementing machine learning methods due to computational limitations, NCDC facilitates such analyses by providing a Python-compatible environment through a remote virtual machine. It is expected to run not only prediction models, but also various causal inference models to find out the relationship between treatment, demographic or lab test variables and subsequent diseases or mortality.

## STRENGTHS AND WEAKNESSES

Compared to Korean health insurance claims [9,23], the sample cohorts are specialized for cancer research. These datasets have detailed information on cancer and its screening [24,25]. Additionally, a sample representing approximately 21% of the data was selected to ensure representativeness. As the data are longitudinal, researchers can track all medical utilization and prognosis before and after cancer onset, as well as analyze the time-to-event for mortality, treatment outcomes, comorbidity conditions after cancer diagnosis, and risk factors of cancer incidence. In Korea, all medical services can be tracked through health insurance claim data because of a single national health insurance system. Moreover, as the sample cohorts are pre-established, the data usage application process is simplified, allowing researchers to access and analyze the data remotely.

This study has few limitations. The database does not include the general population without cancer as a control group; however, it will be updated in the future. Although approximately 20% of the sample satisfied the statistical representativeness, the absolute sample sizes were small. As the sample cohorts allow remote access, the higher the sampling percentage, the greater the possibility of personal identification. Hence, the number of samples cannot be increased to maintain security. However, compared to that in the existing cancer-related cohort data [26], the number of participants in our cancer sample cohorts is not small [27-29]. Therefore, our cancer sample cohorts are suitable for conducting general cancer research. The sample size might limit data analysis when considering secondary cancers, detailed comorbidities, or rare populations. In such cases, researchers can use customized data from CPLD. Data on uninsured treatments and medications, cosmetic procedures, laboratory tests, clinical information, and the use of over-the-counter medications were excluded.

The concept of the CPLD is similar to that of the National Cancer Institute’s SEER-Medicare–linked database [30]. Compared to the SEER-Medicare cohort, our cohorts lacked hospital information and a patient assessment variable; however, they included not only patients aged >65 years, but also younger patients because of the single-payer system. Korea has only one national health insurance system, which enables comprehensive tracking of each patient’s diagnosis, procedures, and examination history. Consequently, our cohort covered all age groups and health utilization before and after cancer diagnosis [31]. Therefore, our data are representative of the entire population.

In conclusion, the Stomach Cancer Sample Cohort, Breast Cancer Sample Cohort, Colorectal Cancer Sample Cohort, and Liver Cancer Sample Cohort are powerful data sources, providing information on health utilization, demographics, socioeconomic status, detailed cancer information, health checkups, and cancer screening results before and after cancer incidence. These cohorts will facilitate evidence for cancer prevention, diagnosis, treatment, and management. The K-CURE project plans to continuously open sample cohorts for different types of cancer. In 2025, it plans to open sample cohorts for lung and pancreatic cancer, followed by sample cohorts for kidney, cervical, and blood cancers in 2026.

## DATA ACCESSIBILITY

The data can be accessed through the K-CURE portal (https://k-cure.mohw.go.kr/). A person must register on the portal and apply for access to the Cancer Sample Cohorts through the “Application for the Data” menu. Researchers must complete the application forms, research proposals, and institutional review board approval documents. Applications are reviewed by the Review Committee of the NCDC. Subsequently, the data and receipts are provided to the applicant at a fee. Sample cohorts can be accessed remotely through a virtual computer environment. Downloading data offline is impossible.

## Figures and Tables

**Table 1.** Number of samples in the cohorts

Variables	Stomach Cancer Sample Cohort		Breast Cancer Sample Cohort		Colorectal Cancer Sample Cohort		Liver Cancer Sample Cohort	
	Population	Sample cohort	Population	Sample cohort	Population	Sample cohort	Population	Sample cohort
Age (yr)								
0-39	6,741	1,691 (25.1)	20,264	4,178 (20.6)	7,178	1,846 (25.7)	2,418	724 (29.9)
40-49	23,547	5,214 (22.1)	66,292	13,321 (20.1)	21,895	4,837 (22.1)	12,192	2,942 (24.1)
50-59	55,963	11,608 (20.7)	58,075	11,714 (20.2)	59,277	12,011 (20.3)	32,870	7,026 (21.4)
60-69	69,168	14,180 (20.5)	30,659	6,329 (20.6)	70,920	14,181 (20.0)	33,084	7,042 (21.3)
70-79	65,297	13,358 (20.5)	13,830	3,024 (21.9)	68,748	13,729 (20.0)	30,083	6,439 (21.4)
≥80	27,387	5,900 (21.5)	3,787	1,020 (26.9)	33,273	6,881 (20.7)	13,677	3,202 (23.4)
Sex								
Male	167,656	34,542 (20.6)	-	-	158,209	31,948 (20.2)	93,053	19,821 (21.3)
Female	80,447	17,409 (21.6)	192,907	39,586 (20.5)	103,082	21,537 (20.9)	31,271	7,554 (24.2)
Region								
Metropolitan	42,244	8,517 (20.2)	43,230	8,611 (20.2)	51,546	10,110 (19.9)	20,919	4,275 (20.4)
City	62,976	13,741 (21.8)	50,337	10,607 (21.8)	63,939	13,383 (21.1)	32,258	7,367 (22.8)
Rural	142,883	29,693 (20.8)	99,340	20,368 (20.8)	145,806	29,992 (20.5)	71,147	15,733 (22.2)
SEER summary stage								
In situ	10,295	2,360 (22.9)	29,304	6,035 (20.6)	29,193	6,201 (21.2)	-	-
Localized	150,082	30,346 (20.2)	95,762	19,280 (20.1)	88,999	18,038 (20.3)	56,618	11,949 (21.1)
Regional	48,703	10,440 (21.4)	54,542	11,151 (20.4)	93,026	18,848 (20.3)	29,937	6,672 (22.3)
Distant	25,881	5,976 (23.1)	8,010	2,011 (25.1)	36,349	7,764 (21.4)	19,255	4,561 (23.7)
Unknown	13,142	2,829 (21.5)	5,289	1,109 (21.0)	13,724	2,634 (19.2)	18,514	4,193 (22.6)
Incidence year								
2012	31,317	6,456 (20.6)	19,263	3,950 (20.5)	31,604	6,393 (20.2)	15,715	3,400 (21.6)
2013	31,061	6,447 (20.8)	20,268	4,164 (20.5)	31,171	6,332 (20.3)	15,656	3,413 (21.8)
2014	30,981	6,505 (21.0)	21,400	4,426 (20.7)	31,141	6,402 (20.6)	15,574	3,433 (22.0)
2015	30,360	6,381 (21.0)	22,502	4,638 (20.6)	31,628	6,495 (20.5)	15,623	3,451 (22.1)
2016	31,674	6,648 (21.0)	25,722	5,264 (20.5)	33,698	6,901 (20.5)	15,643	3,466 (22.2)
2017	31,060	6,545 (21.1)	26,477	5,426 (20.5)	33,793	6,942 (20.5)	15,307	3,390 (22.1)
2018	30,752	6,479 (21.1)	27,852	5,703 (20.5)	33,727	6,913 (20.5)	15,515	3,430 (22.1)
2019	30,898	6,490 (21.0)	29,423	6,015 (20.4)	34,529	7,107 (20.6)	15,291	3,392 (22.2)

Values are presented as number or number (%). SEER, Surveillance, Epidemiology and End Results.

**Table 2.** Frequencies and medical costs by the type of cancer and age group

Type of cancer	Age (yr)	Population		Sample cohorts	
		n	Medical cost (USD)^1^	n	Medical cost (USD)^1^
Stomach	0-39	6,741	9,398±8,320	1,691	9,604±7,955
	40-49	23,547	7,869±7,588	5,214	7,893±7,400
	50-59	55,963	8,194±8,401	11,608	8,188±7,609
	60-69	69,168	8,562±9,849	14,180	8,588±9,604
	70-79	65,297	9,397±10,511	13,358	9,270±9,941
	≥80	27,387	9,623±11,504	5,900	9,672±11,711
Breast	0-39	20,264	10,380±7,716	4,178	10,391±7,725
	40-49	66,292	10,074±7,800	13,321	10,112±7,801
	50-59	58,075	11,297±9,291	11,714	11,341±9,381
	60-69	30,659	11,501±9,156	6,329	11,431±8,577
	70-79	13,830	10,999±10,368	3,024	10,997±10,521
	≥80	3,787	9,487±13,238	1,020	9,288±12,448
Colorectal	0-39	7,178	9,901±10,971	1,846	10,014±11,604
	40-49	21,895	10,585±11,147	4,837	10,421±10,975
	50-59	59,277	10,738±11,741	12,011	10,570±11,324
	60-69	70,920	11,545±12,005	14,181	11,394±11,497
	70-79	68,748	12,605±13,067	13,729	12,425±12,853
	≥80	33,273	12,723±14,438	6,881	12,474±13,657
Liver	0-39	2,418	14,030±13,838	724	13,765±13,926
	40-49	12,192	13,581±16,928	2,942	12,923±15,101
	50-59	32,870	13,558±15,303	7,026	13,316±14,218
	60-69	33,084	13,327±15,902	7,042	13,218±18,131
	70-79	30,083	12,372±11,945	6,439	12,421±11,611
	≥80	13,677	10,197±10,829	3,202	10,133±11,667

Values are preseented as mean±standard deviation.

1 Total medical costs in the cancer incidence year; 1 US dollar (USD)=1,300 Korean won.

**Table 3.** Major variables in the sample cohorts

Cohorts	Domains	Variables	Year
Cancer registry (SMPL_RGST)	Demographical factors	Sex, age at cancer diagnosis	2012-2019
	Cancer registry information	Year and month of first diagnosis, ICD-10, ICD-O-3 (topography), ICD-O-3 (morphology), treatment (surgery, chemotherapy, radiotherapy, immunotherapy, or hormone therapy) within 4 mo after diagnosis, diagnosis method, stages	2012-2019
Cause-of-death data (SMPL_DEATH)	Death	Year and month of death, causes of death	2012-2021
Insurance eligibility (SMPL_BFC)		Type of health insurance, residential area, deciles of insurance fee, type and grade of disability registered	2012-2022
Health insurance claim data	Details of health utilization (SMPL_T200)	Type of institution, region code, the type of encounter (inpatient/ outpatient), main diagnosis code, sub diagnosis code, department code, visit date, the length of stay, total days of prescription, total numbers of medication in prescription, the amount of medical costs, surgery, work-related injury, benefit extension, results of treatment code, and route through hospitalization	2012-2022
	Details of treatment (SMPL_T300)	Procedure codes, therapeutic material codes, medication codes in hospital, the type of treatment, amount of single dose, amount of daily dose or frequency, and total days or frequency	
	Details of diagnosis (SMPL_T400)	Diagnosis code, type of disease code, and department code	
	Details of prescriptions (SMPL_T530)	Prescription ID, medication code in prescription, the amount of single dose, the amount of daily dose, and total days or frequency	
General health checkup	Questionnaire	Past medical history (stroke, cardiac infarction/angina, hypertension, diabetes, and other diseases including cancer), family history (stroke, cardiac infarction/angina, hypertension, diabetes, dyslipidemia, tuberculosis, and other diseases including cancer), smoking (including e-cigarettes), drinking, and physical activity	2012-2022
	Results of examination	Height, weight, body mass index, waist circumference, blood pressure, urine protein, urine glucose, urine occult blood, hemoglobin, fasting blood glucose, cholesterol (total, high-density lipoprotein, and low-density lipoprotein), triglyceride, serum creatinine, SGOT (AST), SGPT (ALT), gamma-GTP	
Cancer screening	Questionnaire	Past cancer treatment history and family history (stomach, breast, colorectal, liver and others), experience about cancer test (UGIS or endoscopy, mammography, FOBT, colonoscopy or double contrast barium enema, pap smear, liver ultrasonography, and chest CT), and past medical history (about stomach, colorectal, liver, lung, and cervix uteri)	2012-2022
	Results of examination	Comprehensive evaluation result, and history of each cancer	
		Stomach cancer screening: UGIS or endoscopy	
		Breast cancer screening: mammography	
		Colorectal cancer screening: FOBT, colonoscopy, and double contrast barium enema	
		Liver cancer screening: liver ultrasonography and serum alpha-fetoprotein test	

ICD-10, International Classification of Diseases-10; ICD-O-3, International Classification of Diseases for Oncology-3; AST, aspartate aminotransferase; ALT, alanine aminotransferase; SGOT, serum glutamic oxaloacetic transaminase; SGPT, serum glutamic pyruvate transaminase; GTP, glutamyl transpeptidase; UGIS, upper gastrointestinal series; FOBT, fecal occult blood test; CT, computed tomography.
